# Use of long acting injectable aripiprazole before and through pregnancy in bipolar disorder: a case report

**DOI:** 10.1186/s40360-019-0330-x

**Published:** 2019-08-28

**Authors:** Isabel Ballester-Gracia, Miriam Pérez-Almarcha, Ana Galvez-Llompart, Miguel Hernandez-Viadel

**Affiliations:** 1 Department of Psychiatry, Clinical Hospital of Valencia, Valencia, Spain; 2 Department of Clinical Medicine, School of Medicine, University of Valencia, Valencia, Spain; 3 Department of Mental Health, INCLIVA Health Research Institute, Valencia, Clinical Hospital of Valencia, Valencia, Spain

**Keywords:** Antipsychotic, Long-acting injectable aripiprazole, Pregnancy, Bipolar disorder

## Abstract

**Background:**

Long-acting injectable (LAI) antipsychotics for psychotic disorders provide advantages in treatment compliance, but data on their use in pregnancy are very limited. We present a clinical case of aripiprazole LAI use in pregnancy.

**Case presentation:**

A 43-year-old woman diagnosed with bipolar disorder, with several relapses due to treatment interruption while trying to conceive. Finally, aripiprazole LAI treatment was planned by mutual agreement between doctor and the patient, who took aripiprazole LAI before and during pregnancy. She gave birth at 40 weeks to a 3500 g baby girl with no congenital malformations, who was healthy at 5 months after delivery.

**Conclusion:**

As far as we know, this is the first case report on aripiprazole LAI use during pregnancy. Although we exercise caution in drawing conclusions from a single case, and each case should be weighed up individually, aripiprazole LAI could be a therapeutic option in similar circumstances.

## Background

Pregnancy, childbirth, postpartum and breastfeeding can be highly stressful periods for women, in both biological and psychosocial terms. Physical and hormonal changes experienced are sometimes accompanied by socio-economic changes.

For women diagnosed with bipolar disorder, the desire to have children implies an additional challenge. Recent studies indicate that a subgroup of women with bipolar disorder could be more sensitive to illness relapse during both pregnancy and postpartum [1]. Besides this, mother and fetus can sometimes face additional risks directly related to adherence or not to the pharmacologic treatment for this illness.

Childbearing is a personal decision, however, and each woman must consider her particular situation at that moment. Adequate and accurate information and planning are essential for all women generally, and particularly for those affected by a serious mental disorder such as bipolar disorder.

On the other hand, the decision of whether to continue with bipolar disorder treatment during pregnancy or not is controversial; the possible teratogenic risk of psychotropic drugs must be balanced against the negative repercussions that a depressive or manic relapse could have on the fetus or newborn child. It has been shown that exposure to lithium during the first trimester of pregnancy entails a moderately increased risk of cardiac malformations in children [2]. Intrauterine exposure to valproic acid has high teratogenic potential in children, who have a higher risk for congenital malformations such as neural tube defects, facial dysmorphism and cleft lip, as well as neurodevelopmental disorders (intellectual disability, ADHD and autism spectrum disorders) [3]. Carbamazepine has also been linked to teratogenic problems [4]. Antipsychotics have not generally been associated with a significant increase in teratogenic risk when used during pregnancy. Although some data reflect a slight increase of teratogenic risk when using phenothiazines, typical antipsychotic drugs are considered relatively safe during this period [5]. Atypical antipsychotics have been linked to metabolic issues and fetus macrosomia when used during pregnancy, but not to an increased risk of fetal malformations [6]. As an example, a prospective cohort study involving 81 women who were exposed to aripiprazole during embryogenesis found that the drug was associated with fetal growth retardation and prematurity, but not with a significantly higher rate of major malformations, spontaneous abortion or gestational diabetes [7].

Information available on the use of long-acting injectable (LAI) antipsychotics during pregnancy is very limited. General recommendations in planned pregnancies are to switch from LAI antipsychotic to oral administration. If a woman becomes pregnant during LAI antipsychotic treatment, recommendations vary between maintaining LAI treatment or switching to oral form.

Of course, each case must be individualized, taking into account the pros and cons of each treatment mode in order to select the best option for the patient. Using the oral form implies a lower teratogenic/perinatal risk, but is also linked to an increased risk of relapse due to treatment abandonment, a less likely occurrence when using LAI treatment.

The aim of this paper is to conduct a literature review of LAI antipsychotic use during pregnancy in order to gain further insight regarding LAI aripiprazole use during this period. We also present a case report with therapeutic interventions, follow up and outcomes.

On July 6 2019 we performed a PubMed, Embase and Web of Science search using the following keywords: long-acting injectable antipsychotics, long-acting injectable risperidone, long-acting injectable paliperidone, long-acting injectable olanzapine, long-acting injectable aripiprazole, antipsychotic monthly, risperidone monthly, paliperidone monthly, olanzapine monthly, aripiprazole monthly, pregnancy. Our literature search focused exclusively on publications on long-acting antipsychotics during pregnancy.

## Case presentation

We treated a 43-year-old woman with history of bipolar disorder whose family history included a first-degree relative with the same illness who had committed suicide 15 years before.

Her symptoms began at the age of 21, with a manic and psychotic episode related to drug abuse (cannabis and cocaine). Throughout the years she has experienced numerous relapses, requiring hospitalization on more than 10 occasions.

The patient’s first contact with our clinic was in 2012 after admission to the Acute Care Psychiatric Unit. She presented with a manic episode and serious behavioral disorder (aggressiveness) and legal issues. She had previously been stable for 4 years, taking low doses of oral olanzapine (2.5 mg/d). After hospital discharge, she was prescribed 800 mg/d amisulpride, 600 mg/d valproate, 0.5 mg/d clonazepam and 4 mg/d biperiden.

Our patient remained stable without any incidents until 2013, when she admitted having discontinued her medication the previous month believing to be pregnant, which turned out to be a false alarm. We explained to her the risk of relapse without adequate medical treatment. However, she came off her medication once more and was admitted again in the Acute Care Psychiatric Unit (2014) because of a manic relapse. After hospital discharge, she followed a treatment plan similar to the one prescribed after the previous admission.

During outpatient follow-up she expressed a desire to conceive. She had had a stable partner for 10 years, despite some relationship crises and break-ups. We gave her the appropriate information regarding therapy options, and explained the need to remove valproate. We informed her about the option of lithium, considering on balance that the possibility of bipolar disorder relapse and consequent risk to mother and baby was greater than teratogenicity, including lithium cardiac risks for the baby. Also, lithium could be a better option than antipsychotics due to protection against manic and depressive relapses. Finally, we agreed to introduce lithium, based on a benefit/risk balance previously discussed with the patient. A few months later she experienced a new manic relapse requiring hospital readmission (2015). She admitted that after starting lithium she had read on the prospectus that taking it was not recommended during the first trimester of pregnancy so she changed her mind and stopped medication again as a precaution in case she was pregnant.

A few months later, she presented with 3-month amenorrhea and a negative pregnancy test. Secondary hyperprolactinemia due to amisulpride was suspected (levels of 363 ng/mL), so we switched to paliperidone. Prolactin levels were decreased to 129 ng/ml.

In view of a history of repeated severe manic relapses because of treatment noncompliance and the patient’s firm desire to conceive we agreed to prescribe LAI paliperidone (100 mg/month). She showed a good clinical response and remained stable. Nevertheless, she gained weight due to the medication, feeling deeply uncomfortable about it. After considering other treatment options, we agreed to stop paliperidone (November 2015) and start LAI Aripiprazole (400 mg/month). She progressed favorably, with good tolerance of medication and normalization of prolactin levels.

In March 2018, she came to the clinic at 2–3 weeks pregnant. The pros and cons of continuing aripiprazole treatment during pregnancy were explained. Based on current evidence, antipsychotics in general and aripiprazole in particular had a low risk of side effects, but potential toxicity could not be completely ruled out. On the other hand, medication discontinuation implied a risk of relapse. She was given information about the side effects of aripiprazole during pregnancy and was provided with instructions to obtain further data (fact sheets from the MotherToBaby website, pharmacy reports from Vall d’Hebron Hospital in Barcelona). After consulting with her partner, she decided to continue treatment, but requested a lower dose. We decreased the LAI aripiprazole dosage to 300 mg/month and agreed with her that she was to come to the emergency room if any warning symptoms were noticed.

As it was considered a high obstetric risk pregnancy, ultrasound controls were performed on weeks 16, 17, 21, 26, 31, 35 and 38, with weekly follow-up by the midwife. Obstetric controls revealed no fetus malformations nor development issues. Pregnancy progressed without complications. She continued to comply with the agreed treatment throughout the whole pregnancy, without recurrence of her illness or significant mood fluctuations.

She gave birth in November 2018 at the gestational age of 40 weeks+ 4 days by spontaneous vaginal delivery, assisted by the midwife without complications. The newborn girl weighed 3500 g, with an Apgar score of 9/10/10, and umbilical cord pH of 7.29. No congenital malformations at birth or development abnormalities were observed at five months after delivery.

Two days after hospital discharge, she came to the clinic as an outpatient and was euthymic, so we agreed to reintroduce the recommended LAI aripiprazole dose of 400 mg/4 weeks.

## Discussion and conclusions

We obtained 35 articles from the literature search, of which only five were relevant to the topic.

Finally, we added another article suggested by a peer reviewer that hadn’t appeared in our search [8]. The six articles were case reports referring to use of LAI antipsychotics during pregnancy.

One case used LAI risperidone [9], two cases LAI paliperidone [8, 10], one case LAI olanzapine [11], another used zuclopenthixol decanoate [12] and the last used LAI aripiprazole [13] but in this case, only the first month of pregnancy. None of the six published cases reported congenital malformations in children.

Bipolar Disorder is a severe mental illness usually diagnosed between 18 and 30 years of age, affecting a stage of life in which a high number of women become pregnant.

Treatment discontinuation during the months before conception can increase the risk of relapse during pregnancy [14], which also can indirectly affect fetus development or increase the possibility of stillbirth, due to various probable consequences for the mother, such as a deterioration in routine and behavior, inadequate nutrition, greater risk of exposure to alcohol or other drugs and poor obstetric monitoring. [15].

Taken together these factors highlight the importance of treatment in bipolar disorder both for the mother and the fetus, given their extreme vulnerability during pregnancy.

In the case presented above, discussing the pros and cons of medication with the patient led to her agreement to follow a treatment plan compatible with her firm and upheld commitment to becoming a mother. Although accepting taking lithium at first, she later changed her mind due to fear of potential harm to the fetus. Finally, LAI aripiprazole was prescribed at a minimum dose throughout the pregnancy (300 mg/4 weeks) and there was no relapse. Labor proceeded without complications and the outcome was a baby girl who is currently 5 months old, healthy and with no congenital malformation nor development issues detected.

As far as we know, this is the first prospective case monitoring the entire pregnancy of a woman with bipolar disorder treated with aripiprazole LAI. The favorable outcome of this case suggests that although there is no definitive evidence on reproductive safety, aripiprazole LAI may be a rational option for pregnant women with bipolar disorder when the expected benefits outweigh the potential risks. Despite the difficulty of research in this field, however, further information is needed.

## Data Availability

Not applicable. All information has been provided by the clinical history and patient interview.

## Abbreviation

LAI: Long-acting antipsychotic
